# Fully automated determination of the cervical vertebrae maturation stages using deep learning with directional filters

**DOI:** 10.1371/journal.pone.0269198

**Published:** 2022-07-01

**Authors:** Salih Furkan Atici, Rashid Ansari, Veerasathpurush Allareddy, Omar Suhaym, Ahmet Enis Cetin, Mohammed H. Elnagar

**Affiliations:** 1 Department of Electrical and Computer Engineering, University of Illinois at Chicago, Chicago, Illinois, United States of America; 2 Department of Orthodontics, College of Dentistry, University of Illinois at Chicago, Chicago, Illinois, United States of America; 3 Department of Oral and Maxillofacial Surgery, College of Dentistry, University of Illinois, College of Dentistry, Chicago, Illinois, United States of America; University of Craiova, ROMANIA

## Abstract

**Introduction:**

We aim to apply deep learning to achieve fully automated detection and classification of the Cervical Vertebrae Maturation (CVM) stages. We propose an innovative custom-designed deep Convolutional Neural Network (CNN) with a built-in set of novel directional filters that highlight the edges of the Cervical Vertebrae in X-ray images.

**Methods:**

A total of 1018 Cephalometric radiographs were labeled and classified according to the Cervical Vertebrae Maturation (CVM) stages. The images were cropped to extract the cervical vertebrae using an Aggregate Channel Features (ACF) object detector. The resulting images were used to train four different Deep Learning (DL) models: our proposed CNN, MobileNetV2, ResNet101, and Xception, together with a set of *tunable* directional edge enhancers. When using MobileNetV2, ResNet101 and Xception, data augmentation is adopted to allow adequate network complexity while avoiding overfitting. The performance of our CNN model was compared with that of MobileNetV2, ResNet101 and Xception with and without the use of directional filters. For validation and performance assessment, k-fold cross-validation, ROC curves, and p-values were used.

**Results:**

The proposed innovative model that uses a CNN preceded with a layer of tunable directional filters achieved a validation accuracy of 84.63%84.63% in CVM stage classification into five classes, exceeding the accuracy achieved with the other DL models investigated. MobileNetV2, ResNet101 and Xception used with directional filters attained accuracies of 78.54%, 74.10%, and 80.86%, respectively. The custom-designed CNN method also achieves 75.11% in six-class CVM stage classification. The effectiveness of the directional filters is reflected in the improved performance attained in the results. If the custom-designed CNN is used without the directional filters, the test accuracy decreases to 80.75%. In the Xception model without the directional filters, the testing accuracy drops slightly to 79.42% in the five-class CVM stage classification.

**Conclusion:**

The proposed model of a custom-designed CNN together with the tunable Directional Filters (CNNDF) is observed to provide higher accuracy than the commonly used pre-trained network models that we investigated in the fully automated determination of the CVM stages.

## I. Introduction

Every individual has a unique growth and development pattern; there are periods of accelerated and decelerated growth [1, 2]. The success of orthodontic/orthopedic treatment depends on optimal treatment timing, especially in addressing craniofacial skeletal imbalances. The optimal treatment timing relies on identifying craniofacial skeletal maturity stages besides the periods of favorable growth for structures such as mandibular condyles and circum-maxillary sutures [3–5]. The chronological age is not reliable enough to reflect the actual circumpubertal growth spurt as it is influenced by gender, ethnicity, genetic profile, and nutrition. Bone age assessment using radiographic analyses was reported to be more accurate than chronological age in determining skeletal maturation, growth rate, the peak period of growth, and the remaining growth potential [6–9]. Hand-wrist radiographs have been a reliable method for assessing skeletal maturity; however, they require extra radiation exposure [10, 11].

Cervical vertebra maturation (CVM) staging in lateral cephalometric radiographs is an alternative method to determine skeletal maturation, as lateral cephalometric radiography is routinely required for orthodontic diagnosis and treatment planning in orthodontic practice with no additional radiographs required to assess the CVM stages [2, 12]. The validity and reliability of the CVM staging besides its correlation with the hand-wrist method (HWM), have been supported by multiple studies [10, 12, 13]. The major limitation of the CVM method is that it is not user-friendly and needs experienced practitioners; researchers reported poor reproducibility among nonexpert examiners [14]. In contrast, another group reported a satisfactory level of reliability after specific training on the visual assessment of the CVM stages [15, 16]. Another limitation is that the conventional visual assessment of CVM stages is not integrated with the computerized cephalometric analysis. There have been recent attempts to develop a computer-assisted classifier; however, the proposed methods require the clinician to identify 19 or 26 digital landmarks on the cervical vertebra, which still needs to be done manually, and the accuracy of the classification depends on the identification of these landmarks [17–19].

The use of Machine-Learning (ML) techniques in the field of medical imaging is rapidly evolving, and a fully automated diagnostic approach has gained attention with its promise for reducing human error as well as the time and effort needed for the task [20, 21]. Recently, an advanced ML method called “deep learning (DL)” has become the most sought-after tool to solve image pattern recognition and classification problems and has been extensively used in the medical field [20]. The application of DL to study human growth and development from radiographs is a promising idea that needs to be explored. The present study aims to apply DL to develop a fully automated high-performance system to detect and classify the CVM stages.

The key original contributions of this paper are as follows:

- The custom-designed convolutional neural network (CNNDF) incorporates directional filters as an initial layer to emphasize the edges of Cervical Vertebrae.
- The convolutional directional filter layer parameters are also updated during the training process.
- The CNNDF has fewer parameters than the pre-trained networks (MobileNetV2, ResNet101 and Xception) and it does not require data augmentation.
- The directional filter layer improves the classification accuracy of CVM stages not only in the proposed CNNDF but also in pre-trained networks and the classical SVM method.

## II. Material and methods

The data set used in developing our algorithm consists of digitized images of scanned lateral cephalometric films for subjects aged between 4 and 29 obtained from the American Association of Orthodontists Foundation (AAOF) Craniofacial Growth Legacy Collections, an open data source. Films from Michigan, Bolton Brush, and Iowa Facial Growth Studies were used in this study [22]. The AAOF legacy collection was collected from growth studies, so they are longitudinal records. Since we used an open-source dataset, the present study was granted IRB exempt status (2021–0480) by the Office of Human Subjects Protection at The University of Illinois at Chicago. All the films were downloaded as jpg files using Firefox FTP software (Mountain View, Calif) and they do not require any special access privileges to access. All cephalometric radiographs included in this study are of adequate quality, with the second (C2), third (C3), and fourth (C4) cervical vertebrae clearly observable. The exclusion criteria were limited to poor-quality images or malformations in the head and neck region.

The images were studied and labeled by an expert Orthodontist Scientist (MHE) with more than ten years of experience in classifying CVM. Cervical maturation stages were classified into six stages (CS1- CS6) in keeping with the methodology from previous studies [2, 23]. Cervical Stage 1 (CS1): the inferior borders of vertebral bodies C2 to C4 are flat, and the third and fourth cervical bodies are trapezoidal. Cervical Stage 2 (CS2): visible notch along the inferior border of the second cervical vertebra, while the lower borders of the third and fourth vertebral bodies remain flat with both C3 and C4 retaining a trapezoidal shape. Cervical Stage 3 (CS3): visible notching of the inferior borders of C2 and C3; the inferior border of C4 remains flat while the C3 and C4 bodies still retain a trapezoidal shape. Cervical Stage 4 (CS4): the inferior borders of vertebral bodies C2 to C4 have obvious concavities along their inferior surfaces, with the shapes of C3 and C4 bodies being horizontally elongated rectangular rather than trapezoidal. Cervical Stage 5 (CS5): differentiated from CS 4 based on the shapes of C3 and/or C4, with these body shapes becoming square. All three cervical bodies have notches. Cervical Stage 6 (CS6): at least one of the third and fourth cervical bodies has assumed a vertically elongated rectangular morphology. The length of the posterior border is longer than the inferior border. Besides, the cortical bone appears better delineated at CS 6 than at CS 5.

The main data set was classified into six stages of CVM (CS1-CS6) [2, 12, 23]. Another improved method of the CVM assessment introduced by Baccetti et al. [24] classified the CVM into five stages (CVMS I- CVMS V). In the five-stage classification, the CS1 and CS2 stages were merged into a single stage referred to as CVMS I (the lower borders of all the three vertebrae are flat, with the possible exception of a concavity at the lower border of C2). Furthermore, CS3 is denoted as CVMS II. We assessed our model in performing classification into both five CVM stages (CVMS I- CVMS V) and six CVM stages (CS1-CS6). In this paper, we examine both the six-stage and five-stage classification problems.

The principal evaluator (MHE) repeated the classification process two weeks later, and the intra-examiner reproducibility for the cervical vertebral stages was tested by weighted kappa (wk). The intra-examiner agreement was almost perfect (wk = 0.95). Furthermore, another evaluator, an Oral and Maxillofacial surgeon (OS), performed the repeated classification process, and the inter-examiner agreement was strong (wk = 0.90). The final data set of 1018 classified images by the principal evaluator (MHE) was used in this study based on the MHE’s greater experience. In our data set, the number of lateral cephalograms belonging to cervical stages CS1, CS2, CS3, CS4, CS5, and CS6 were 154, 187, 174, 159, 167, and 177, respectively. In CVMS classification, CS1 and CS2 classes are merged to form the CVMS I class. CVMS II is the same as CS3, CVMS III is the same as CS4, and so on.

*Data Augmentation for Pre-trained Networks*: We note that our proposed CNN with directional filter layer does not need data augmentation during training. However, pre-trained deep learning algorithms such as Xception usually require huge training data sets to produce accurate classification results. If the size of the training data set is small, then the Xception model may overfit the training data and achieve high accuracy on the training data set while performing poorly on validation and testing data sets. There are several solutions to avoid overfitting the model to the training data set. These include transfer learning, batch normalization, and data augmentation. Since our data set is not very large, we use data augmentation and transfer learning to increase the number of images in our data set to prevent overfitting in the case of pre-trained networks MobileNetV2, ResNet101, and Xception

To augment the data, new images are created by shifting the RoI images to the right, left, up and down, and by rotating them clockwise and counterclockwise. Our CVM image data set has 1018 images. Of these 1018 images, 761 are used in the training set and 257 in the testing set, respectively. These 257 testing images are set aside to use for the evaluation of the model at the end. By implementing the augmentation methods, we created 10 additional images out of each image in the training set. A total of 761×11 = 8371 images is generated using augmentation for training. The density distribution of the classes is preserved after the augmentation. The augmented data set is used as a training set only for the pre-trained deep learning algorithms. Again, we emphasize that the proposed CNN with directional filter layer does not need data augmentation during training.

The data set contains labeled images a large subset of which serves as a training set needed to develop a deep learning model. Since the edges of the vertebral body shapes are critical in determining the CVM stage, we decided to investigate a custom-designed innovative deep learning model that includes tunable preprocessing to emphasize the edges in the input image. Our deep learning architecture is based on Convolutional Neural Network (CNN), which is a classifier widely used in image recognition applications. To prepare our data set for training, the images are first segmented to extract the region of interest. The deep learning architecture is augmented with a bank of directional filters aimed at improving the accuracy of the classification task. The results attest to the improvement in accuracy due to the use of the proposed augmented architecture.

In the following subsections, we describe the steps of our lateral cephalogram classification algorithm. We first segment the images to extract the regions of interest using an aggregate channel feature object detector as described in Subsection II-A. The directional filters used in this study are described in Subsection II-B; and finally, the custom designed CNN model is delineated in Subsection II-C.

### A. Image RoI segmentation

We first segment the image and identify the spine (Cervical Vertebrae) region using the so-called Aggregate Channel Features (ACF) object detector [25]. It is a classical computer vision method that analyzes a given image in sliding windows using image feature pyramids. This avoids the process of manually cropping the spine region in each image in the database. The use of the ACF object detector helps automate the process of preparing images for subsequent analysis with the deep learning algorithm, thereby making the overall processing computationally more efficient. As a result, the skull, jaw, and irrelevant background regions are removed before the images are applied to the deep learning algorithm. The ACF object detector automatically extracts the Region of Interest (RoI) in the images thereby reducing the search space of the deep learning structure.

We trained the ACF object detector using 300 images and applied it to extract relevant RoI sub-images from the 1018 larger images in our data set. The ACF detector correctly identified the RoIs in 703 out of the remaining 718 images, yielding an accuracy of 98%. In the remaining 15 images, we manually cropped the ROI before feeding it to our proposed CNNDF model and pre-trained MobileNetV2, ResNet101, and Xception models. Because all the segmented images have variable size, they are resized to a common size of 77x35. We note that resizing the images can be a sensitive operation because it might change the relative dimensions of the vertebrae. This may impact classification as the fundamental difference between CVM stages is the size and the curvature of the vertebrae in X-ray images. Therefore, the resizing should be done without changing the aspect ratio of the image. Next, instead of feeding the resized ROI images directly to the DL networks, the edges of vertebral bodies in the ROI images are emphasized using eight directional filters described in [26, 27]. The outputs of directional filters are fed to the DL networks. In fact, we make the directional filters the first layer of the DL networks and even update their parameters during the network training process making the layer parameters tunable.

We review the directional filters in the next subsection.

### B. Directional filters

Deep learning structures contain many convolutional filters whose weights are learned during training. These convolutional filters are initialized with random numbers in general. In this section, we introduce an initial layer consisting of eight directional filters. We initialize these filters in such a way that they highlight the edges of X-ray images in eight directions. We design the initial values of the filters using a Fourier domain method. During the standard learning process, we also allow the network to update the values of the front-end layer filters. Outputs of directional filters provide multi-channel inputs to the rest of the deep CNN. Since we have eight directional filters, we have more input layers than the commonly used three input layers. In the pre-trained models we investigated, we first use a stage of directional filtering following which we adhere to the available model of the pre-trained network. In designing the directional filters, we focused on using simple low-order filters that work adequately for the task. Higher-order filters produce better frequency characteristics but may cause ringing artifacts at edges in an image.

We used a one-dimensional high-pass prototype filter obtained from a 7-th order half-band Lagrangian maximally flat lowpass filter with the following transfer function to design the directional filters [28, 29]:

$$H_{lp}\left(z\right)=1+\frac{9}{16}\left(z^{1}+z^{-1}\right)-\frac{1}{16}\left(z^{3}+z^{-3}\right),$$ (1)

with a DC gain of *H*_*lp*_ (*e*^*j*0^) = *H*_*lp*_ (1) = 2.

The high-pass filter is obtained with the transformation *H*_*hp*_ (*z*) = *H*_*lp*_ (−*z*), and the corresponding impulse response of the high-pass filter is

$$h_{hp}\left[n\right]=\left\{\frac{1}{16},0,\frac{-9}{16},1,\frac{-9}{16},0,\frac{1}{16}\right\}.$$ (2)

**Fig 1:**
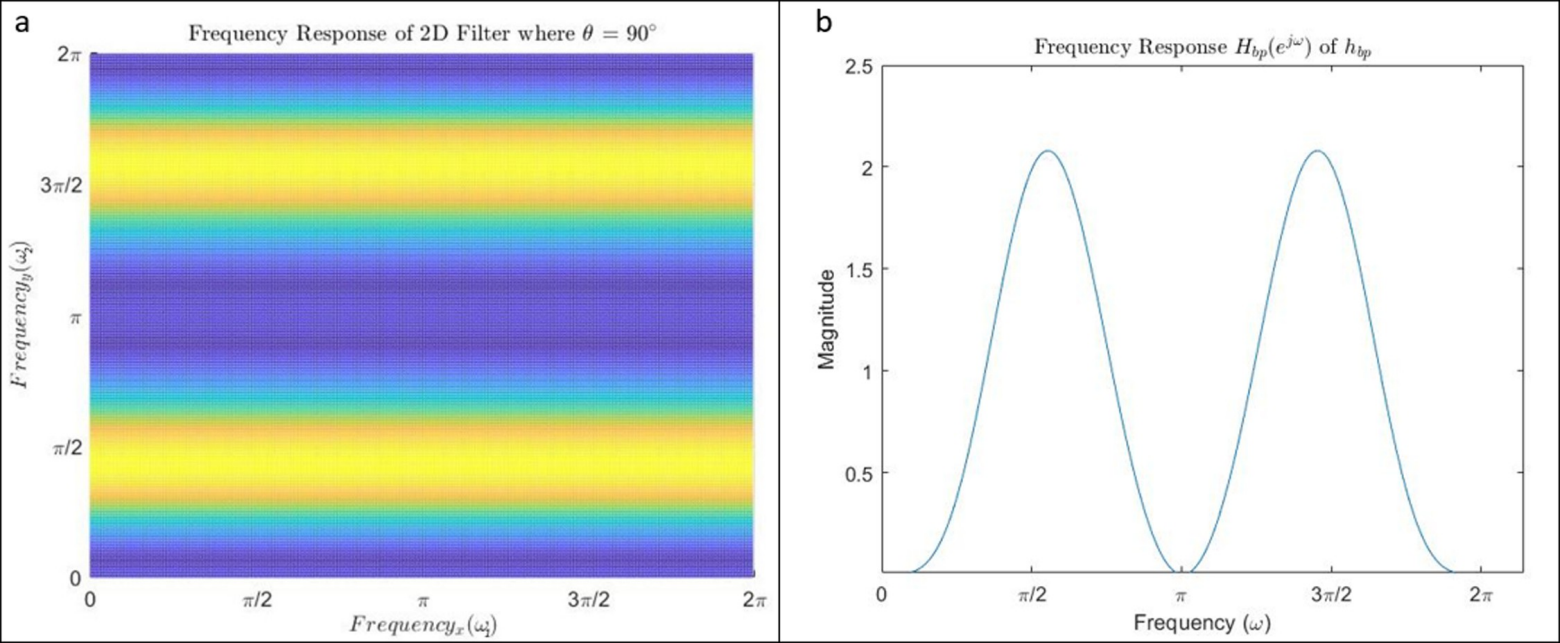
(a) The frequency response *H*_*bp*_ (*e*^*jω*^) of *h*_*bp*_. The filter has gain of 2 at $\omega =\frac{\pi }{2}$ and 0 at *ω* = 0 and π, exhibiting a band-pass response. (b)The frequency response *H*_*θ*_ of the vertical edge filter. This filter $h_{90^{{}^{\circ}}}\left[n_{1},n_{2}\right]=\delta \left[n_{1}\right]h_{bp}\left[n_{2}\right]$. It has a magnitude peak at $\omega_{2}=\frac{\pi }{2}$.

This filter is concatenated with a lowpass filter with impulse response *h*_*c*_ [*n*] = {1,2,1} to avoid the amplification of noise at high frequencies and the overall frequency response is that of a band-pass filter:

$$h_{bp}\left[n\right]=\left(h_{c}*h_{hp}\right)\left[n\right],$$ (3)

where * represents the convolution operation. The frequency response *H*_*bp*_ (*e*^*jω*^) of the filter with impulse response *h*_*bp*_ is shown in Fig 1A. The impulse response $h_{0^{{}^{\circ}}}$ of the prototype (horizontal) two-dimensional (2-D) filter obtained from *h*_*bp*_ is:

$$h_{0^{{}^{\circ}}}\left[n_{1},n_{2}\right]=h_{bp}\left[n_{1}\right]\delta \left[n_{2}\right]$$ (4)

The 2-D impulse response obtained by rotating $h_{0^{{}^{\circ}}}$ by an angle *θ* degrees is denoted by $h_{\theta^{{}^{\circ}}}$. The filter with impulse response $h_{0^{{}^{\circ}}}$ produces zero-crossings at the location of the vertical edges. We rotate the impulse response $h_{0^{{}^{\circ}}}$ by *θ* = −63.43°, −45°, −26.56°, 0°, 26.56°, 45°, 63.43°, 90° to obtain eight filters. Due to our choice of low-order filters, the lower density of samples in the diagonal direction produces multi-band responses in the case of *θ* = 45° rather than the bandpass responses observed in the case of *θ* = 0° or 90°. The frequency response for *θ* = 90° is shown in Fig 1B. For example, the filter corresponding to 45° has the impulse response

$$h_{45^{{}^{\circ}}}\left[n_{1},n_{2}\right]=\left\{\begin{array}{cc} h_{bp}\left[n_{1}\right] & if\;n_{1}=n_{2}\\ 0 & otherwise \end{array}\right.$$

which produces zero-crossings corresponding to 45° edges.

The coefficients of eight filters are provided in the Supporting information section. The frequency responses of the 2-D filters are shown in Supporting information section. The coefficients of the directional filters are obtained using the method described in [26]. Instead of rotating the coefficients of the horizontal filter $h_{0^{{}^{\circ}}}$ using the bilinear interpolation, the authors propose a method resulting a sparse set of filter coefficients whose sums along the *θ* = {±26.56° ±90, ±45° ±90, ±63.43° ±90} are approximately equal to the coefficients of the horizontal filter. The filter with *θ* = 90 is simply the vertical version of the horizontal filter. The 2-D filter impulse responses corresponding to *θ* = {0°, ±26.56°, ±45°, ±63.43°, 90°} are also available in [26].

**Fig 2:**
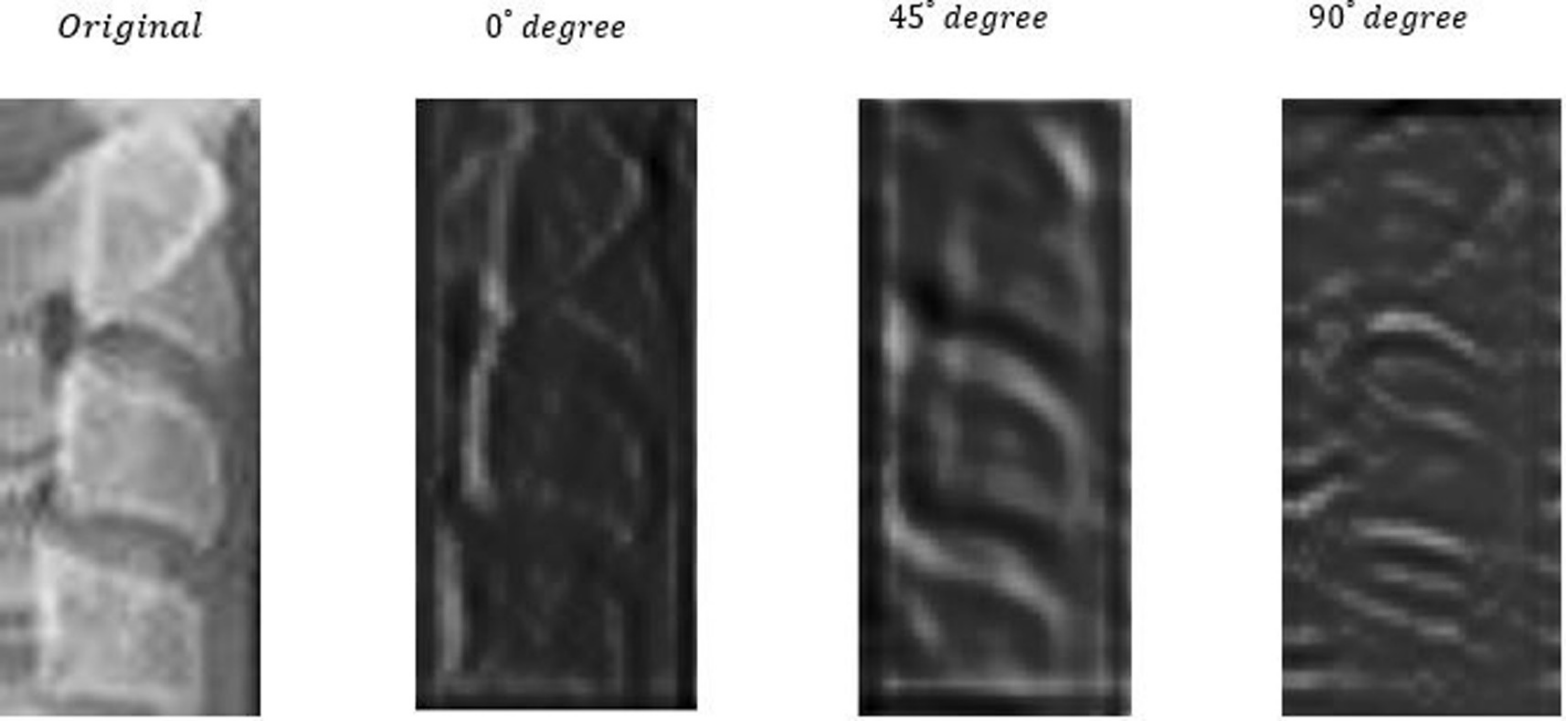
Effect of the directional filters on a ROI of a cervical vertebrae image (leftmost image). Outputs of 0°, 45°, and 90° filters are shown in the middle left, middle right, and the rightmost figures, respectively.

The motivation behind using the directional filters is to start the deep learning model using our prior domain knowledge. Since the images among the classes are not dramatically different as in natural images, we estimate that highlighting the edges of the bones more than two directions will give an advantage to the deep network model. We illustrate the effect of the directional filters on an instance of cervical vertebrae in Fig 2. These observations motivated our use of the directional filters (DF) in the proposed method. The performance accuracy with and without directional filters is examined in Section III.

As a result, eight different channels are created at the output of the directional filters. These filters are implemented with direct convolution. Outputs of these filters serve as initial estimates in our networks. Instead of starting the initial layer with random numbers, we start the training process with the directional filters emphasizing edges at different angles.

### C. Convolutional Neural Network with Directional Filter (CNNDF) layer

The most efficient and common trainable model to classify the images into a specific number of classes is the Convolutional Neural Network (CNN) [30, 31]. Fundamentally, convolution layers are designed to extract the information from the images using a 2-D convolution operation. The channels created after the convolution layers can contain vital information regarding the class, and the channels are emphasized or de-emphasized accordingly. In this project, initially, we decided to use a custom CNN with residual learning to measure the capacity of a DL model on our data set. More elaborate models–MobileNetV2 [32], ResNet101 [33], and Xception [34] are also used for the purpose of comparison. These pre-trained networks are trained on ImageNet [35] and their performance are presented in ImageNet Challenge [36].

To determine how many layers to use, we try to strike a balance between overfitting and underfitting. It is common knowledge that there is always a chance that the model may overfit due to the data set characteristics or the complexity of the model. To achieve better balance, it is recommended to start from a simple CNN model, then increase model complexity as needed for better results. Using this strategy, 6 convolutional layers are found to be best suited for our classification task. However, due to the nature of the convolution layers, the problem of vanishing/exploding gradients might arise as the CNN gets deeper. Residual learning is introduced into the model as skip connection blocks to avoid the vanishing gradient problem [33]. In addition to the regular convolution layers, a convolution layer with no activation function and kernel size (1,1) is used to match the filter numbers in the so-called skip connection block. Batch normalization and dropout layers are key mechanisms adopted after the regular convolution layers to avoid overfitting as the model trains. The non-linear activation function *“ReLU”* is adopted after the batch normalization layers. The outputs of these two convolution layers are added to achieve the final output. Maximum pooling layers are used to decrease the number of parameters that the model must learn. The architecture of the proposed CNN is given in Table 1. The first layer consists of directional filters described in Section II.B. We believe that the directional filters help highlight the details and the differences of the classes through the layers.

**Table 1:** (a) The architecture of our CNN with Directional Filter (CNNDF) layer, and (b) details of the skip connection block.

(a)		
Layer Name (type)(kernel size)(filters)	Output Shape	Connected to
Input (input layer)	77 x 35 x 1	
Directional (conv) (7x7)(8)	77 x 35 x 8	Input
Match (DepthwiseConv) (7x7)(8)	77 x 35 x 8	Directional
Dir Leaky Relu (Leaky ReLU)	77 x 35 x 8	Match
Conv_0 (conv) (1x1)(8)	77 x 35 x 8	Input
Batch_0 (batch normalization)	77 x 35 x 8	Conv_0
ADD_1 (add)	77 x 35 x 8	DirLeakyRelu& Batch_0
Max_1 (max pooling 2d)	39 x 18 x 8	ADD_1
Skip_Con_1(skip connection)(N/A)(32)	39 x 18 x 32	Max_1
Max_2 (max pooling 2d)	20 x 9 x 32	Skip_Con_1
Skip_Con_2(skip connection)(N/A)(64)	20 x 9 x 64	Max_2
Max_3 (max pooling 2d)	10 x 5 x 64	Skip_Con_2
Skip_Con_3(skip connection)(N/A)(128)	10 x 5 x 128	Max_3
Max_4 (max pooling 2d)	5 x 3 x 128	Skip_Con_3
Skip_Con_4(skip connection)(N/A)(256)	5 x 3 x 256	Max_4
Flatten (Flatten)	3840	Skip_Con_4
Dense_1 (Dense)	64	Flatten
Batch_11 (batch normalization)	64	Dense_1
Relu_11 (ReLU)	64	Batch_11
Output_Dense (Dense) (Softmax)	5	Relu_11
(b) Skip Connection Block		
Layer Name (type) (kernel size)(filters)	Connected to	
Conv_no (conv) (3x3) (filter no)	Max_no	
Batch_no (batch normalization)	Conv_no	
Relu_no (ReLU)	Batch_no	
Dropout_no (dropout)	Relu_no	
Conv_skip_no (conv) (1x1) (filter no)	Max_no	
Batch_skip_no (batch normalization)	Conv_skip_no	
ADD_no (add)	Dropout_no & Batch_skip_no	

As pointed out above, the initial layer of the CNN consists of directional filters which are initialized using the filter weights described in Section II.B. During training we also tuned the directional filters by updating their weights using the backpropagation algorithm. Instead of initializing these filters with random numbers, we initialize them with coefficients that can detect edges in different directions. In other words, we use our domain knowledge to guide the network to achieve the goal of recognizing CVM stages.

**Fig 3:**
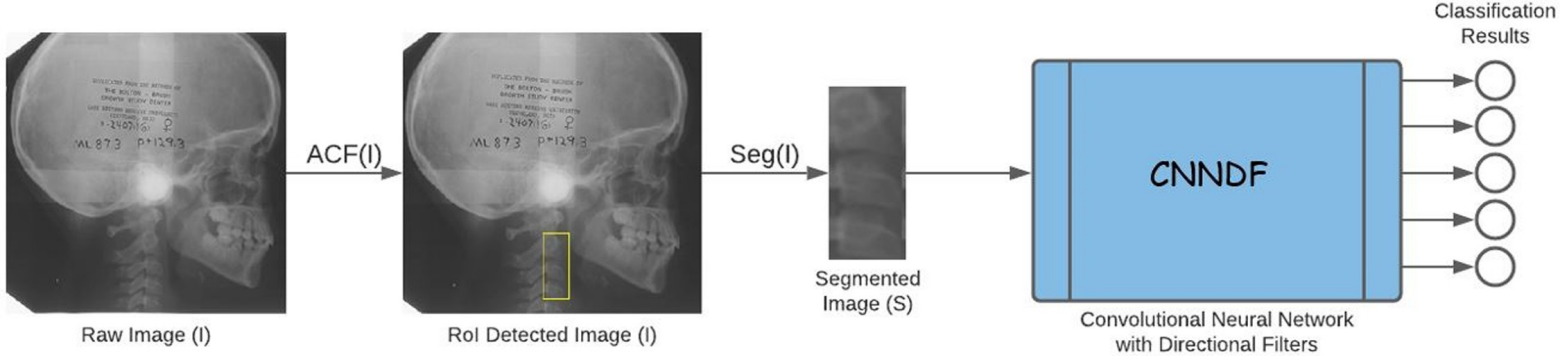
The overview of our image processing pipeline: Given an input raw image *I*, we detect the RoI using ACF object detector, *I = ACF(I)*, extract the RoI segment using cropping, *S =* Seg(*I*), and feed the cropped image into the network to obtain the classification results.

The choice of the model components discussed define the final structure of our model. The overview of our model is given in Fig 3. To find the optimal components, a series of deep learning models and pre-processing techniques are used. To compare the results; MobileNetV2, ResNet101, Xception, and our own fully connected CNN are used as benchmarks with various directional filter choices.

## III. Results and comparisons

**Fig 4:**
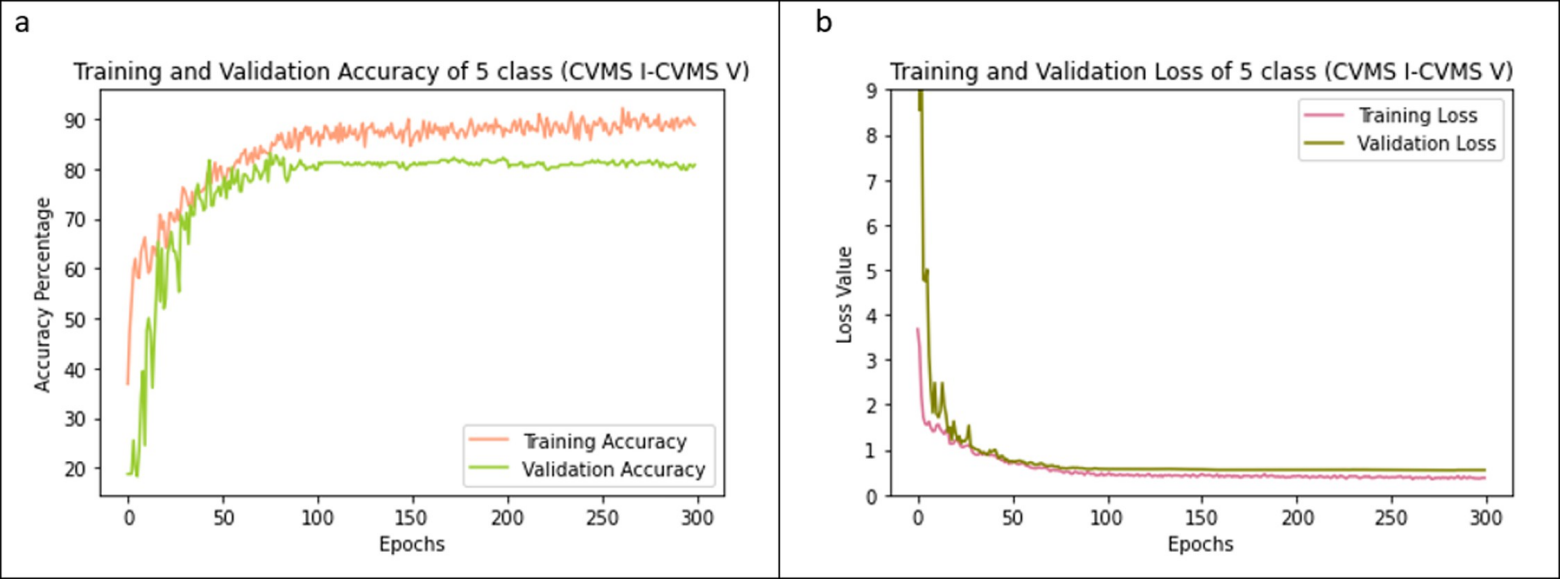
Training and validation accuracy and losses for 5-stage classification. The accuracies (a) and losses (b) of CNN model with the directional filters obtained by averaging 5 folds are shown.

**Fig 5:**
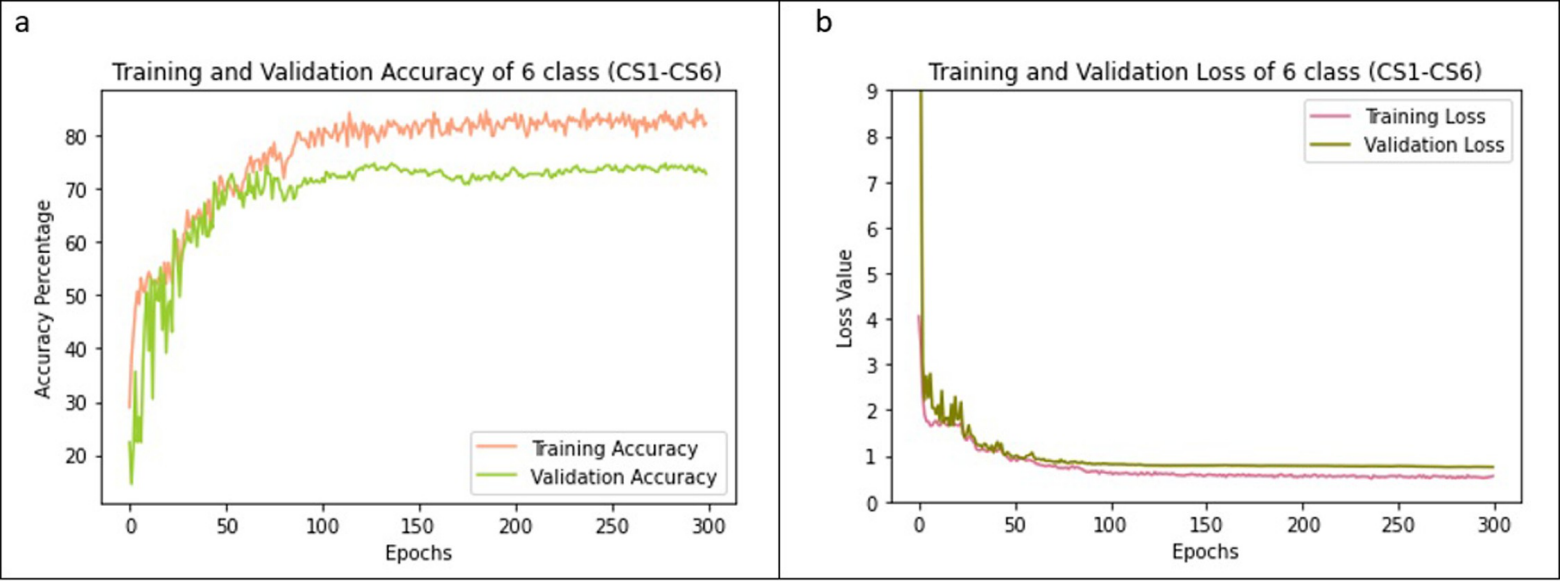
Training and validation accuracy and losses for 6-stage classification. The accuracies (a) and losses (b) of CNN model with the directional filters obtained by averaging 5 folds are shown.

In this section, the results obtained and the comparison between different models with or without the pre-processing are presented. As mentioned earlier, the proposed method yields 75.11% accuracy for six classes (CS1-CS6). It achieves a higher 84.63% validation accuracy in 5-stage classification (CVMS I-CVMS V). The average accuracy attained and loss incurred in k-fold cross validation for classification into 5 stages are shown in Fig 4A and 4B, respectively. For comparison, the k-fold (k = 5) cross validation results for 6-stage classification over the epochs are depicted in Fig 5A and 5B. In this paper, 5-fold cross-validation is used to ensure that the model is applicable to any arbitrary data set. In every fold the testing data set and training data sets are different showing that the model works on any arbitrary testing data set. A training set which contains 761 images is used. We choose a 4:1 splitting in the 5-fold cross validation. Images are split *before* the augmentation, and then the augmentation is applied. Therefore, there is no overlap between the training fold and validation fold in any part of the training process. We do not use any data augmentation during the training of the proposed CNN model with directional filters. Data augmentation is used only to train pre-trained models (MobileNetV2, ResNet101 and Xception).

As seen from the Figs 4 and 5, the model performs better on 5-stage classification than on 6-stage classification. This difference should be interpreted with a closer look at the two-class classification for CS1 and CS2. It is observed that the difference between images of CS1 and CS2 is the curvature in vertebrae in CS2 which is not a strong differentiator and makes the error in distinguishing CS1 and CS2 high. This is the key reason why the accuracy is lower for 6-stage classification compared with 5-stage classification (CVMS I-CVMS II). We found that classification based on Baccetti et al.’s 5-stage CVM classification [24] provides more reliable results in all the networks that we investigated.

**Fig 6:**
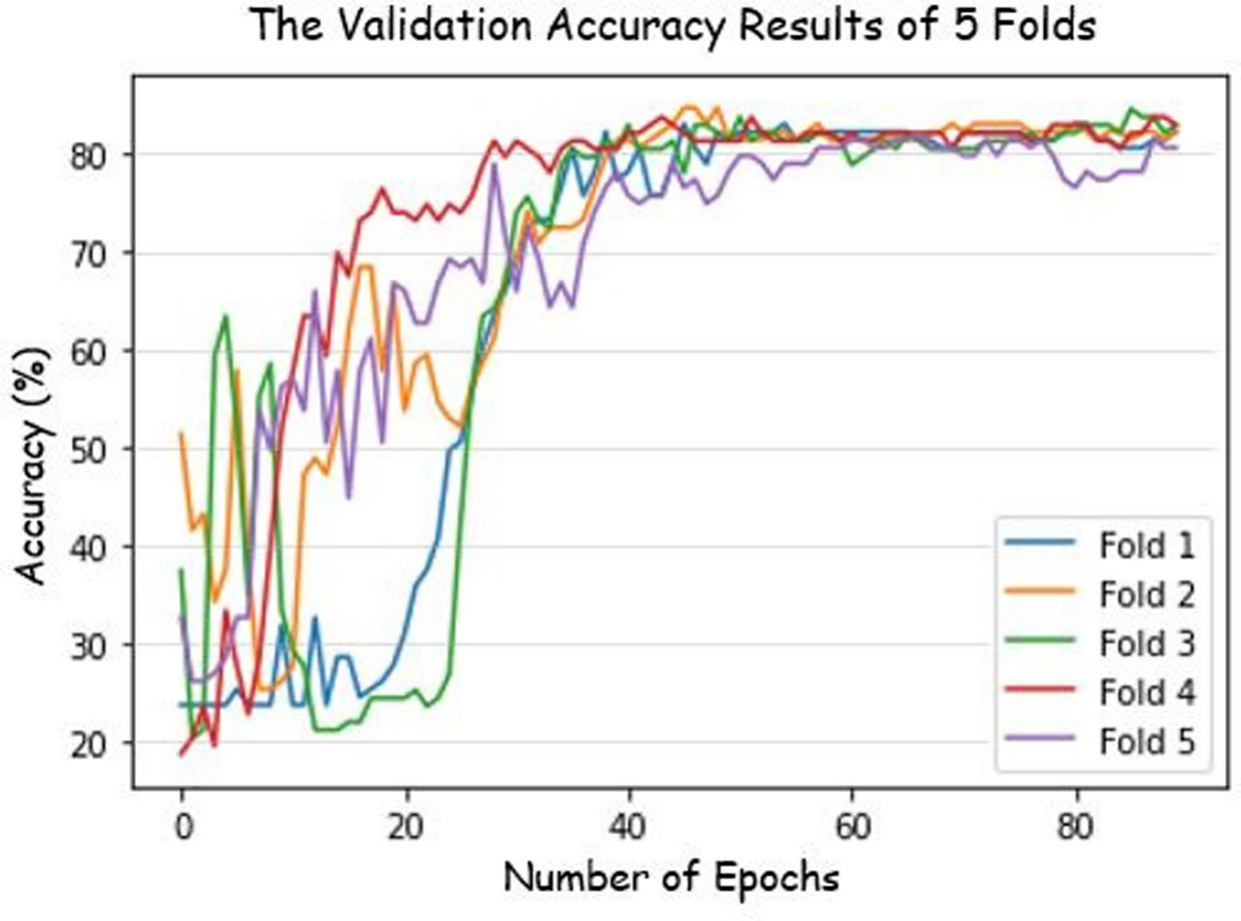
Validation accuracy of our custom CNN model with the directional filters over 5 folds. Five different folds were used for testing the model. The behaviors of the trainings are consistent with one another. The final accuracy is above 83%.

The confusion between CS1 and CS2 originates primarily but not entirely from the curvature difference between C1 and C2. The posture of the subjects also influences the decision. If an X-ray image obtained from a CS1 subject is inclined slightly downwards, there is a chance that this image is predicted as CS2 because of the posture. As a result, Baccetti et al.’s 5-stage classification is also adopted for classification in this paper. The detailed validation accuracy results of every fold are shown in Fig 6. The accuracy percentage is validated in every fold, which confirms the validity of the model.

**Fig 7:**
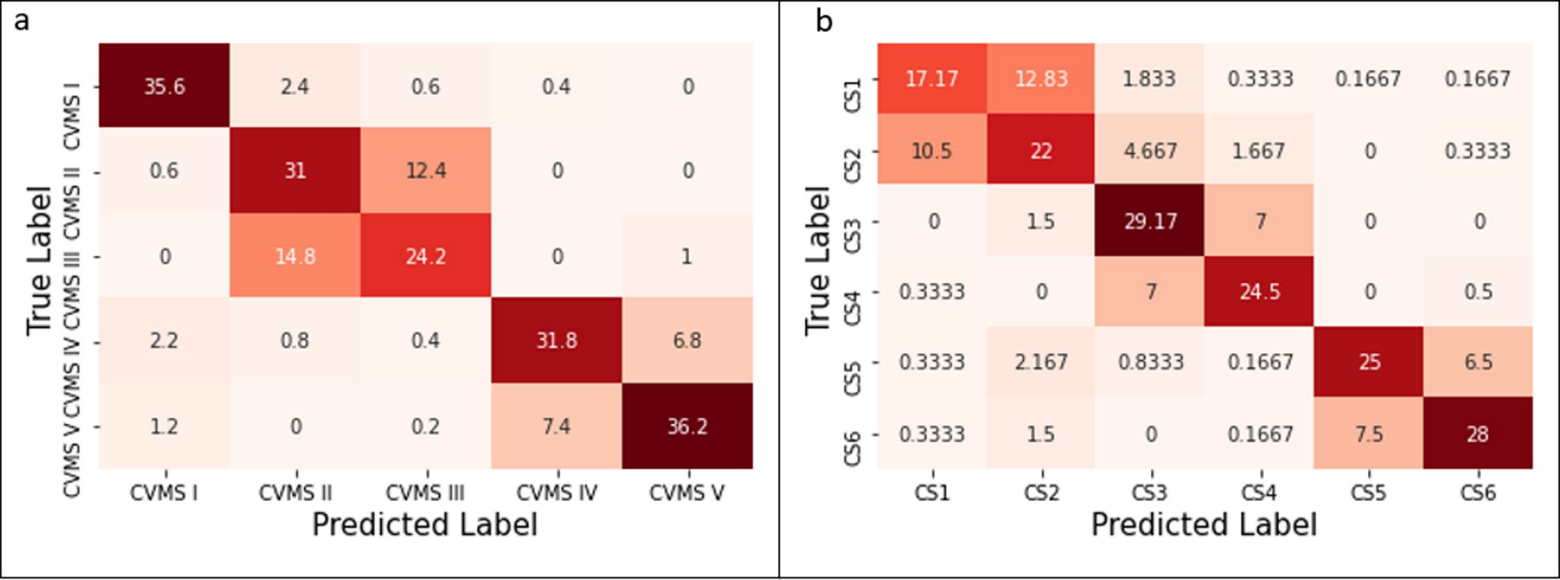
Average confusion matrices after fivefold testing for (a) 5-stage classification and (b) 6-stage classification are shown.

To evaluate the model on the testing data set, the confusion matrices of both 5-class and 6-class problems are shown in Fig 7. Since the model is validated over 5 folds, 5 different confusion matrices are obtained per classification and averaged. The average confusion matrix of 5-stage classification is shown in Fig 7A. For example, we had 39 CVMS I images in our test set. We correctly predicted 35.6 of these images (non-integer value is obtained due to averaging of 5 experiments); 2.4 of 39 images are classified as CVMS II; 0.6 images are predicted as CVMS III, 0.4 images are classified as CVMS IV, respectively. Other rows of the matrices are obtained in a similar manner. In the case of 6-stage classification, CS1 and CS2 classes have the lowest accuracy as shown in the first two rows of the confusion matrix in Fig 7B. We observe that the proposed model makes most of the misclassifications between neighboring classes.

To further evaluate the proposed method and possibly improve the model, we compare our method with other pre-trained models. MobileNetV2 [32], ResNet101 [33] and Xception [34] networks are used for comparison. In addition, a classical approach, Support-Vector Machine (SVM) with a Radial Basis Function (RBF) kernel is used for comparison.

To see the effect of using directional filters, we first implemented all the models without the Directional Filters (DF). As shown in Table 2, the accuracy of the model that uses the CNNDF is higher than that of models that do not use DFs in both 5-class and 6-class classifications, which emphasizes the impact of the directional filters.

**Table 2:** The accuracy comparison of the various ML models investigated. Every ML model was tested with and without the directional filters. The results are shown in a table for comparison. Fully connected CNN with the Directional Filters (CNNDF) outperforms every other combination.

Classes	Models Filters	MobileNetV2	ResNet101	Xception	SVM	CNNDF
**5 Classes** (CVMS I–CVMS V)	*With Directional Filters*	78.54%	74.10%	80.86%	69.85%	84.63%
	*Without Directional Filters*	76.01%	72.40%	79.42%	71.68%	80.75%
**6 Classes** (CS1 –CS6)	*With Directional Filters*	69.92%	68.64%	71.44%	60.21%	75.11%
	*Without Directional Filters*	66.98%	66.85%	70.71%	59.05%	72.37%

The best result with 84.63% accuracy is obtained in 5-stage classification problem with our custom CNNDF model. Resnet101 and MobileNetV2 with the 8 directional filters do not reach even 80% accuracy, achieving 74.10% and 78.54%, respectively, in 5-stage CVM classification. The well-known Xception network [34] derived from InceptionV3 [37] yields 80.86% accuracy, with performance closest to our CNNDF. Our CNNDF clearly outperforms other well-known image classification networks in terms of the accuracy of the classification results. The pre-trained models are developed to classify natural scene images and objects; their use in medical image analysis may not be as effective in classifying natural scenes. The other significant result from Table 2 is the classification result for 6-stage classification. The best performance is obtained when our CNNDF is used (75.1% accuracy). Moreover, our CNNDF has only 715 K parameters. MobileNetV2, the simplest algorithm among the DL networks that we considered, has 2.2 M parameters. As a result, CNNDF is also the most efficient in terms of the number of trainable parameters and it does not require any data augmentation. The other networks are much larger in size, and they require data augmentation for training. The SVM with the RBF kernel produces inferior results compared with the deep learning based methods and much lower accuracy than that of CNNDF.

**Fig 8:**
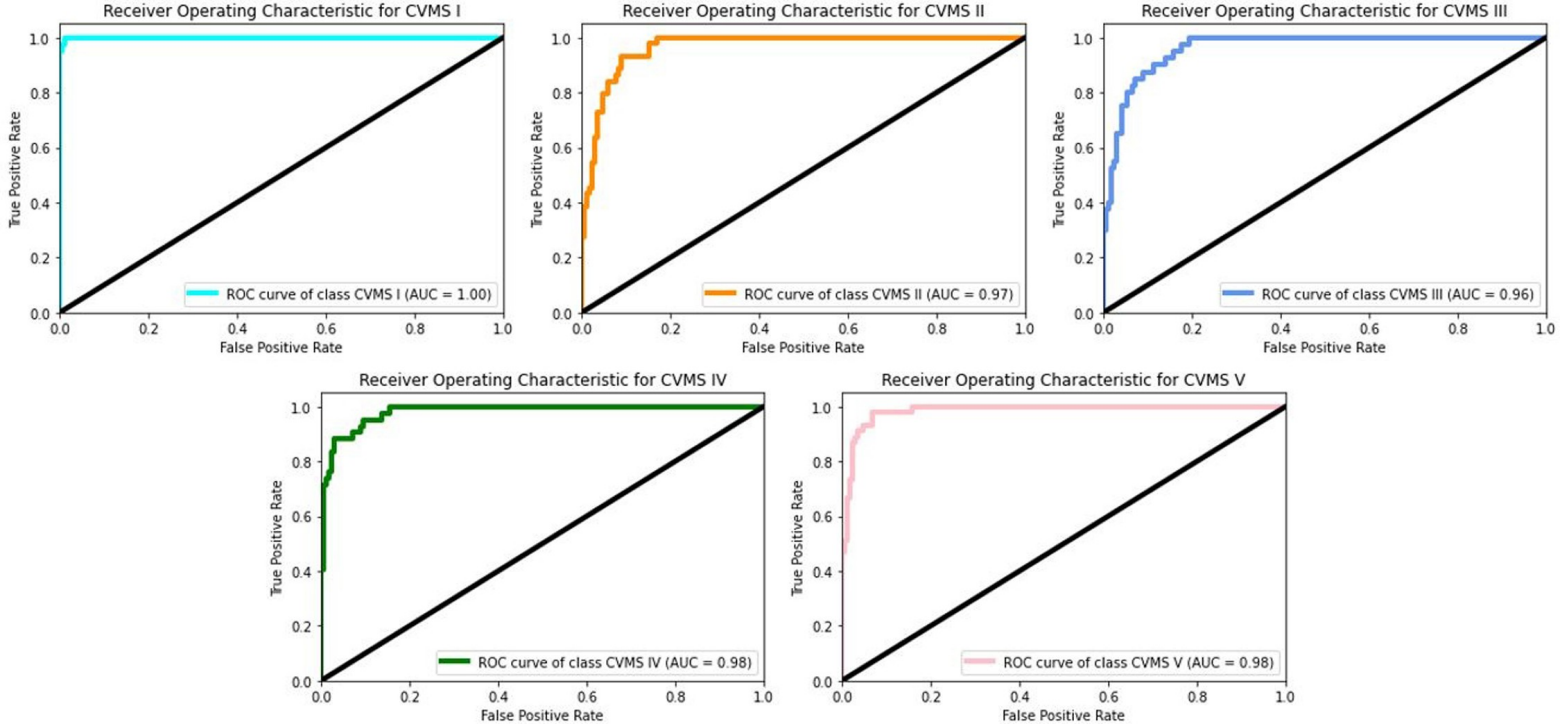
ROC curves and the corresponding AUC values. The curves are plotted TPR vs FPR one selected class against the remaining classes.

Receiver Operating Characteristic (ROC) curve is a tool to measure the performance of a given ML model. It utilizes two different parameters: True Positive Rate (TPR) and False Positive Rate (FPR). These two parameters are defined as:

$$TPR=\frac{TP}{TP+FN}\;FPR=\frac{FP}{FP+TN}$$

where TP, FP, FN, TN are True Positive, False Positive, False Negative, and True Negative values, respectively. The ROC is a plot of TPR vs FPR at different threshold levels to show how well the model performs the given classification task. A related measure to assess the performance of the model is the Area Under Curve (AUC) derived from ROC. AUC assumes a value between 0 and 1, which a value close to 1 indicates that a reliable performance is achieved by the model. ROC can be generalized and extended to multiple classes using “one class vs rest” strategy, where one selected class is labeled as Class 1 while the remaining classes are together labeled as Class 0. This process is repeated for all five classes; therefore, five different ROCs are obtained. Based on these ROCs, the corresponding AUCs are calculated. The ROC plots and the corresponding AUC values are given in Fig 8.

It is also worth noting that although our proposed model achieves 84.6% accuracy, the AUC values are all above 0.9. This is due to the adoption of “one vs rest” methodology. Although the model functions as the multi-class classifier, the ROC curves are obtained by using two alternate classes created by “one vs rest” distinction.

In addition to AUC values, precision, recall and F1 score values of 5-class classification and 6-class classification are given in Table 3A and 3B, respectively. Table 3A and 3B show that 5-stage classifier is effective in classifying the stages.

**Table 3:** Performance metrics of the classes for both classification task. Recall, Precision and F1 Score are calculated for every class in both classification tasks. 5-stage classification (a) contains higher values than 6-stage classification (b).

a			
**Metrics** **Class**	**Recall**	**Precision**	**F1 score**
*CVM I*	0.913	0.899	0.906
*CVM II*	0.633	0.705	0.667
*CVM III*	0.640	0.605	0.622
*CVM IV*	0.803	0.757	0.779
*CVM V*	0.804	0.823	0.813
b			
**Metrics** **Class**	**Recall**	**Precision**	**F1 score**
*CS1*	0.528	0.599	0.561
*CS2*	0.562	0.550	0.556
*CS3*	0.774	0.671	0.719
*CS4*	0.758	0.724	0.741
*CS5*	0.685	0.765	0.723
*CS6*	0.747	0.789	0.767

The impact of the directional filters are clearly visible in the fully connected CNN with seven convolutional layers because the recognition accuracy significantly increased from 80.75% to 84.63%. To establish the statistical significance of the improvement due to the use of DF, the p-value is calculated. If the p-value is smaller than the alpha level, a defined threshold for p-value chosen here to be 0.01, we reject the observed findings as arising from the null hypothesis. The null hypothesis (*H*_*0*_) is that the observed accuracy of the fully connected CNN model does not improve with the use of DF, whereas the alternative (*H*_1_) is that the CNN model performance improves with the DF.

To obtain the p-value, the testing data set is divided into 7 subsets to increase the sample number. Each subset contains 30 images. The image distribution is set up according to the initial density of the classes. A normal distribution is fitted to the subset accuracy histograms for both hypotheses, and its properties are calculated. The means of the normal distributions of null and alternative hypotheses were computed as 0.778 and 0.826, respectively. Also, the standard deviations of two distributions are calculated as 0.0762 and 0.0633, respectively, and the p-value is much smaller than the alpha level of 0.01. To conclude, the null hypothesis is rejected with this p-value, i.e., the alternative *H*_1_, that the CNN model with the directional filters improves performance, is accepted. With the strong evidence obtained from the accuracy results and the p-value, we state that the use of the directional filters improved the performance since it helps the model with a prior knowledge on the difference among the classes.

## IV. Discussion

The most common skeletal maturity indicators are skeletal maturation of hand and wrist and Cervical Vertebrae Maturation (CVM) stages. The hand-wrist method (HWM) has not changed significantly since Greulich and Pyle (GP) published their atlas in 1950. GP method compares the patient’s radiograph with an atlas of representative ages and determines the bone age [38]. Another scoring method introduced by Tanner-Whitehouse (TW2) is based on examining 20 specific bones [39]; both methods are tedious, and for orthodontic practice, time-consuming; besides it needs additional radiation exposure [40, 41]. Evaluation of CVM stages on the lateral cephalometric radiograph is preferred in clinical practice because it eliminates additional radiography [2]. However, the CVM stages methods are influenced by experience and training [42, 43]. The AAOF collection was used to develop the CVM classification methods and to study the correlation between CVM development stages and growth. It is significant to state that no previous studies have been published specifically for applying Artificial Intelligence to CVM development staging using the AAOF legacy collection.

With the recent advances in AI, the automated diagnosis and interpretation of medical images are rapidly evolving aiming to increase accuracy, decrease variability and eliminate the tediousness of the task [44]. Determination of the growth and development by AI is a field that needs to be explored. This study aimed to develop a fully automated pipeline for the determination of the CVM stages.

There have been a few attempts to develop computer-assisted methods to determine CVM stages; Baptista et al. developed a semi-automated method for CVM in 2011 [18]. They located and measured the 20 landmarks on every cephalogram. They constructed three classification models based on Naive Bayes (NB) and achieved 90% with one stage deviation. Their study showed us that using a pattern classification method can help orthodontics identify the CVM staging and achieve high accuracy. Amasya et al. compared different ML models on the CVM degrees classification [19]. Their model requires manual labeling and marking of 26 landmarks on the cervical vertebrae for manual extraction of the image feature; then ML clinical decision support system used the numerical values of the features to determine the CVM stage. They have developed five different ML models to compare the results; Logistic Regression (LR), Decision tree (DT), Random Forest (RF), Artificial neural network (ANN), Support vector machine (SVM). Similarly, Kok et al. defined 19 reference points on the cervical vertebrae, took 20 different linear measurements and compared seven different algorithms: k-nearest neighbors(k-NN), NB, DT, ANN, SVM, LR, and RF [17]. Both studies have concluded that the most stable and accurate performance is achieved when ANN is used as the main model. This may lead us to the ANN’s impact on the classification task; therefore, using a deep learning model on the determination of the CVM degree on the lateral cephalograms is a decent idea.

Until the work of Makaremi et al., the use of a DL model to determine the six CVM stages has not been investigated. They have proposed a DL model to determine the cervical vertebrae maturation degree using only an entropy filter [45]. Their model consists of two convolution layers followed by a max-pooling layer, one convolution layer followed by a max-pooling layer, and a fully connected neural network with a hidden layer. The CNN model of [45] achieves an accuracy of 70% on the 6-stage classification in our dataset. We could not make a comparison with the work of Makaremi et al. in their dataset because we do not have access to it. The entropy filter is used to measure the attributes of distributions of the pixel values of X-ray images. However, entropy filters are not trainable in their model. One of the significant aspects of the work by Makaremi et al is to use entropy filters to help the DL model capture the context of the lateral cephalograms. That motivated us to examine the use of directional filters, which emphasize the CV edges in the cephalograms, in aiding the feature selection in subsequent deeper layers of the CNNDF structure.

Other X-ray image classification examples include [41, 46, 47]. Lee et al. constructed a fully automated DL system to analyze the hand, wrist radiographs and perform Bone Age Assessment (BAA), which indicates the growth [41]. They built a complete preprocessing system where they categorize, standardize, segment, and label the X-ray images to prepare the data set. The preprocessed and annotated training data set is fed into a variant of the Inception Network, the Google-Net [46]. Lee et al. attempted to create the first fully automated BAA using a DL model. A similar study is carried out by Tajmir et al. [47].

Our model shows high accuracy in classifying the CVM images into 5 classes (CVMS I-CVMS V), higher than the accuracy of classification into 6 stages. Baccetti et al. classified the CVM into 5 stages based on analysis of 6 consecutive observations of untreated subjects [24]; their findings revealed that no statistically significant discrimination exists between CS 1 and CS 2. The presence of a concavity at the lower border of the second cervical vertebra was not a distinctive feature of CVS 2 when compared with CVS 1 [24]. Therefore, they merged two former prepubertal stages (CS 1 and CS 2) into one stage. This merged Cervical Vertebral Maturation Stage is referred to as CVMS I. The peak in mandibular growth will occur not earlier than one year after this stage; hence the clinician can wait for one year at least for a radiographic re-evaluation to start treatment with a functional appliance [24]. Both six and five stages of CVM classification methods showed a high correlation with the middle phalanx of third finger ossification stages [46]. Classification into both six and five stages are usable in daily clinical practice as both can discriminate between prepubertal, circumpubertal, and post-pubertal stages. [2, 12, 23, 24, 46, 48]. Therefore, BAA can be examined with classification either into 5 different classes (CVMS I-CVMS V) or into 6 classes (CS1-CS6), as measured and compared in this paper. It is noted that a fully connected CNN with 6 convolutional layers model with the directional filters shows good performance in classification into 5 classes, achieving 84.63% accuracy on the testing data set.

The use of directional filters as a preprocessing layer improves the accuracy in CVM classification problem. However, this approach may not be applicable to an arbitrary object recognition problem but is suitable in applications where strengthening edge information aids classification. Our aim is to use our domain knowledge to guide the network by introducing the directional filters which emphasize the edges of the cervical vertebrae. Since the difference between CVM stages are determined according to the edges and curvatures of cervical vertebrae, we use the directional filter based-edge detector layer to improve the performance of the deep neural network. Our CNN with the directional filters (CNNDF) provides better results than pre-trained networks such as Xception. We believe that the strong performance of our method stems from the initial layer of directional filters. We also show that using a trainable preprocessing layer improves the accuracy result in the CVM image classification task. Another aspect of this model is the number of images that we use in our database. We used higher number of images than the Makaremi et al.’s study [45] and our data consisted of longitudinal consecutive cephalometric observations.

## V. Conclusion

This paper presents a deep learning model with novel tunable pre-processing to classify lateral cephalograms into CVM stages. A custom-designed CNNDF model with eight tunable directional filters is introduced. Directional filter layer significantly improved the accuracy of the CNNDF and the other pre-trained networks in CVM stage classification problem. The p-value calculation reveals the significance of performance improvement due to the directional filters. Our experimental results shows that the proposed CNNDF model performs better that other pre-trained DL models. The CNNDF produces the best result compared with the pre-trained MobileNetV2, ResNet101, and Xception models, with or without the directional filters. Our method achieved an 84.63% and 75.1% in five- and six-class CVM stage classification problems, respectively, on the testing data set. The proposed CNNDF model can be used as an effective tool for determining the skeletal maturity stage and treatment timing, especially for clinicians with less experience; furthermore, it could have multiple forensic applications.

## Supporting information

S1 File (DOCX)

## References

[1] Hägg U, Taranger J. Maturation Indicators and the Pubertal Growth Spurt. Am. J. Orhod. 1982;82:299–309. doi: 10.1016/0002-9416(82)90464-x 6961802

[2] Baccetti T, Franchi L, McNamara JA. The Cervical Vertebral Maturation (CVM) method for the Assessment of Optimal Treatment Timing in Dentofacial Orthopedics. Semin Orthod. 2005;11:119–29.

[3] Franchi L, Baccetti T, McNamara JA. Postpubertal Assessment of Treatment Timing for Maxillary Expansion and Protraction Therapy followed by Fixed Appliances. Am. J. Orhod. Dentofacial Orthop. 2004;126:555–68. doi: 10.1016/j.ajodo.2003.10.036 15520688

[4] Angelieri F, Franchi L, Cevidanes LS, McNamara JA. Diagnostic Performance of Skeletal Maturity for the Assessment of Midpalatal Suture Maturation. Am. J. Orthod. Dentofacial Orthop. 2015;148:1010–16. doi: 10.1016/j.ajodo.2015.06.016 26672707

[5] Perinetti G, Primožič J, Franchi L, Contardo L. Treatment effects of removable functional appliances in pre-pubertal and pubertal Class II patients: A systematic review and meta-analysis of controlled studies. PLoS One. 2015;10. doi: 10.1371/journal.pone.0141198 26510187 PMC4624952

[6] Suri S, Prasad C, Tompson B, Lou W. Longitudinal comparison of skeletal age determined by the Greulich and Pyle method and chronologic age in normally growing children, and clinical interpretations for orthodontics. Am. J. Orthod. Dentofacial Orthop. 2013;143:50–60. doi: 10.1016/j.ajodo.2012.08.027 23273360

[7] Björk A, Helm S. Prediction of the age of maximum puberal growth in body height. Angle Orthod. 1967;37:134–143. doi: 10.1043/0003-3219(1967)037<0134:POTAOM>2.0.CO;2 4290545

[8] Franchi L, Baccetti T, De Toffol L, Polimeni A, Cozza P. Phases of the dentition for the assessment of skeletal maturity: A diagnostic performance study. Am. J. Orthod. Dentofacial Orthop. 2008;133:395–400. doi: 10.1016/j.ajodo.2006.02.040 18331939

[9] Mellion ZJ, Behrents RG, Johnston LE. The pattern of facial skeletal growth and its relationship to various common indexes of maturation. Am. J. Orthod. Dentofacial Orthop. 2013;143:845–54. doi: 10.1016/j.ajodo.2013.01.019 23726335

[10] Fishman LS. Radiographic evaluation of skeletal maturation. A clinically oriented method based on hand-wrist films. Angle Orthod. 1982;52:88–112. doi: 10.1043/0003-3219(1982)052<0088:REOSM>2.0.CO;2 6980608

[11] Grave KC, Brown T. Skeletal ossification and the adolescent growth spurt. Am. J. Orthod. 1976;69:611–19. doi: 10.1016/0002-9416(76)90143-3 179326

[12] Hassel B, Farman AG. Skeletal maturation evaluation using cervical vertebrae. Am. J. Orthod. Dentofacial Orthop. 1995;107:58–66. doi: 10.1016/s0889-5406(95)70157-5 7817962

[13] Uysal T, Ramoglu SI, Basciftci FA, Sari Z. Chronologic age and skeletal maturation of the cervical vertebrae and hand-wrist: Is there a relationship? Am. J. Orthod. Dentofacial Orthop. 2006;130:622–28. doi: 10.1016/j.ajodo.2005.01.031 17110259

[14] Nestman TS, Marshall SD, Qian F, Holton N, Franciscus RG, Southard TE. Cervical vertebrae maturation method morphologic criteria: Poor reproducibility. Am. J. Orthod. Dentofacial Orthop. 2011;140:182–8. doi: 10.1016/j.ajodo.2011.04.013 21803255

[15] Rainey BJ, Burnside G, Harrison JE. Reliability of cervical vertebral maturation staging. Am. J. Orthod. Dentofacial Orthop. 2016;150:98–104. doi: 10.1016/j.ajodo.2015.12.013 27364211

[16] Perinetti G, Caprioglio A, Contardo L. Visual assessment of the cervical vertebral maturation stages a study of diagnostic accuracy and repeatability. Angle Orthod. 2014;84:951–6. doi: 10.2319/120913-906.1 24665865 PMC8638509

[17] Kök H, Acilar AM, İzgi MS. Usage and comparison of artificial intelligence algorithms for determination of growth and development by cervical vertebrae stages in orthodontics. Prog Orthod. 2019;20:41. doi: 10.1186/s40510-019-0295-8 31728776 PMC6856254

[18] Baptista RS, Quaglio CL, Mourad LH, Hummel AD, Caetano CA, Ortolani CL, et al. A semi-automated method for bone age assessment using cervical vertebral maturation. Angle Orthod. 2012;82:658–62. doi: 10.2319/070111-425.1 22059467 PMC8845536

[19] Amasya H, Yildirim D, Aydogan T, Kemaloglu N, Orhan K. Cervical vertebral maturation assessment on lateral cephalometric radiographs using artificial intelligence: Comparison of machine learning classifier models. Dentomaxillofac. Radiol. 2020;49. doi: 10.1259/dmfr.20190441 32105499 PMC7333473

[20] Lee JG, Jun S, Cho YW, Lee H, Kim GB, Seo JB, et al. Deep learning in medical imaging: General overview. Korean J. Radiol. 2017;18:570–84. doi: 10.3348/kjr.2017.18.4.570 28670152 PMC5447633

[21] Park JH, Hwang HW, Moon JH, Yu Y, Kim H, Her SB, et al. Automated identification of cephalometric landmarks: Part 1—Comparisons between the latest deep-learning methods YOLOV3 and SSD. Angle Orthod. 2019;89:903–9. doi: 10.2319/022019-127.1 31282738 PMC8109157

[22] Anon. AAOF Legacy Collection Home Page.

[23] McNamara JA, Franchi L. The cervical vertebral maturation method: A user’s guide. Angle Orthod. 2018;88:133–43. doi: 10.2319/111517-787.1 29337631 PMC8312535

[24] Baccetti T, Franchi L, McNamara JA. “An improved version of the cervical vertebral maturation (CVM) method for the assessment of mandibular growth.” Angle Orthod. 2002;72(4):316–23. doi: 10.1043/0003-3219(2002)072<0316:AIVOTC>2.0.CO;2 12169031

[25] Dollar P, Appel R, Belongie S, Perona P. Fast Feature Pyramids for Object Detection. IEEE Trans. Pattern Anal. Mach. Intell. 2014;36:1532–45. doi: 10.1109/TPAMI.2014.2300479 26353336

[26] Bagci AM, Ansari R, Reynolds WD. Low-complexity Implementation of non-subsampled directional filter banks using polyphase representations and generalized separable processing. IEEE Int. Conf. Electro Inf. Technol. 2007;422–7.

[27] Bozkurt A, Suhre A, Cetin AE. Multi-scale directional-filtering-based method for follicular lymphoma grading. Signal, Image and Video Process. 2014;8:63–70.

[28] Kim CW, Ansari R, Cetin AE. A class of linear-phase regular biorthogonal wavelets. ICASSP-92: 1992 ICASSP IEEE Int. Conf. Acoust. Speech Signal Process. 1992;4:673–6.

[29] Ansari R, Kim CW, Dedovic M. Structure and design of two-channel filter banks derived from a triplet of halfband filters. IEEE Transactions on Circuits and Systems II: Analog and Digital Signal Processing. 1999;46:1487–96.

[30] LeCun Y, Bengio Y, Hinton G. Deep learning. Nature. 2015;521:436–44. 10.1038/nature14539 26017442

[31] Bengio Y, Lecun Y. Convolutional Networks for Images, Speech, and Time-Series. In: Editor Arbib MA, editor. The Handbook of Brain Theory and Neural Networks. 2^nd^ ed. The MIT Press; 1995. p. 276–279.

[32] Howard AG, Zhu M, Chen B, Kalenichenko D, Wang W, Weyand T, et al. MobileNets: Efficient Convolutional Neural Networks for Mobile Vision Applications. 2017.

[33] He K, Zhang X, Ren S, Sun J. Deep Residual Learning for Image Recognition. Proc. IEEE Comput. Soc. Conf. Comput. Vis. Pattern Recognit. (CVPR). 2016 Jun 26-Jul 1; Las Vegas, USA; 2016. p. 770–8.

[34] Chollet F. Xception: Deep Learning with Depthwise Separable Convolutions. Proc. IEEE Comput. Soc. Conf. Comput. Vis. Pattern Recognit. (CVPR). 2017 Jul 22–26; Honolulu, Hawaii; 2017. p. 1251–8.

[35] Anon. ImageNet. https://www.image-net.org/.

[36] Russakovsky O, Deng J, Su H, Krause J, Satheesh S, Ma S, et al. ImageNet Large Scale Visual Recognition Challenge. Int. J. Comput. Vis. 2015;115:211–52.

[37] Szegedy C, Vanhoucke V, Ioffe S, Shlens J, Wojna Z. Rethinking the Inception Architecture for Computer Vision. Proc. IEEE Comput. Soc. Conf. Comput. Vis. Pattern Recognit. (CVPR). 2016 Jun 26-Jul 1; Las Vegas, USA; 2016. p. 2818–26.

[38] Greulich WW, Idell PS. Radiographic Atlas of Skeletal Development of the Hand and Wrist. Am. J. Med. Sci. 1959;238:393.

[39] Tanner JM, Whitehouse RH, Cameron N. Assessment of Skeletal Maturity and Prediction of Adult Height (TW2 Method). 1989. 10.1080/03014468400007151 6547581

[40] Gaskin CM, Kahn SL, Bertozzi JC, Bunch PM. Skeletal Development of the Hand and Wrist: A Radiographic Atlas and Digital Bone Age Companion. Oxford, UK; Oxford University Press. 2013.

[41] Lee H, Tajmir S, Lee J, Zissen M, Yeshiwas BA, Alkasab TK, et al. Fully Automated Deep Learning System for Bone Age Assessment. J. Digit. Imaging. 2017 Mar 8;30:427–41. doi: 10.1007/s10278-017-9955-8 28275919 PMC5537090

[42] Khajah A, Tadinada A, Allareddy V, Kuo CL, Nanda R, Uribe F, et al. Influence of type of radiograph and levels of experience and training on reproducibility of the cervical vertebral maturation method. Am. J. Orthod. Dentofacial Orthop. 2020 Feb;157(2):228–39. doi: 10.1016/j.ajodo.2019.03.025 32005475

[43] Morris KM, Fields HW, Beck FM, Kim DG. Diagnostic testing of cervical vertebral maturation staging: An independent assessment. Am. J. Orthod. Dentofacial Orthop. 2019 Nov; 156(5): 626–32. doi: 10.1016/j.ajodo.2018.11.016 31677671

[44] Oren O, Gersh B, Bhatt D. Artificial Intelligence in Medical Imaging: Switching from Radiographic Pathological Data to Clinically Meaningful Endpoints. The Lancet Digit. Health. 2020 Sep;2:E486–E488. doi: 10.1016/S2589-7500(20)30160-6 33328116

[45] Makaremi M, Lacaule C, Mohammad-Djafari A. Deep Learning and Artificial Intelligence for the Determination of the Cervical Vertebra Maturation Degree from Lateral Radiography. Entropy. 2019 Dec;21:24.

[46] Szegedy C, Liu W, Jia Y, Sermanet P, Reed S, Anguelov D, et al. Going Deeper with Convolutions. Proc. IEEE Comput. Soc. Conf. Comput. Vis. Pattern Recognit. (CVPR). 2015 Jun 7–12; Boston, MA, USA; 2015. p. 1–9.

[47] Tajmir S, Lee H, Shailam R, Gale HI, Nguyen JC, Westra SJ, et al. Artificial Intelligence-Assisted Interpretation of Bone Age Radiographs Improves Accuracy and Decreases Variability. Skelet. Radiol. 2018;48:275–83. doi: 10.1007/s00256-018-3033-2 30069585

[48] Tikku T, Khanna R, Sachan K, Agrawal S. Correlation of Improved Version of Cervical Vertebral Maturation Indicator with Other Growth Maturity Indicators. J Indian Orthod Soc. 2013 Jan; 47(1): 28–32.

