# Supplementary material for: Fully automated determination of the cervical vertebrae maturation stages using deep learning with directional filters
Source: PLoS One. 2022 Jul 1;17(7):e0269198. doi: 10.1371/journal.pone.0269198 (PMC9249196; doi:10.1371/journal.pone.0269198)

Supporting information

APPENDIX

Here, we present the filter coefficients of the directional filters in S1 Fig. The frequency responses of the eight filters are shown in S2 Fig. In S3 Fig , we show the diagram of the model without the directional filters.

**S1 Fig**. **Filter coefficients of the directional filters in real domain.**


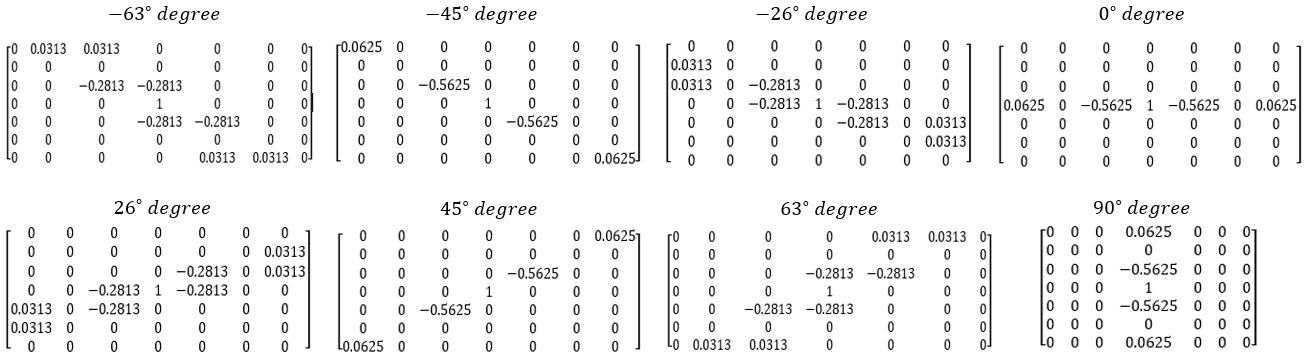


**S2 Fig.** **Frequency responses of the eight directional filters in surface view.**


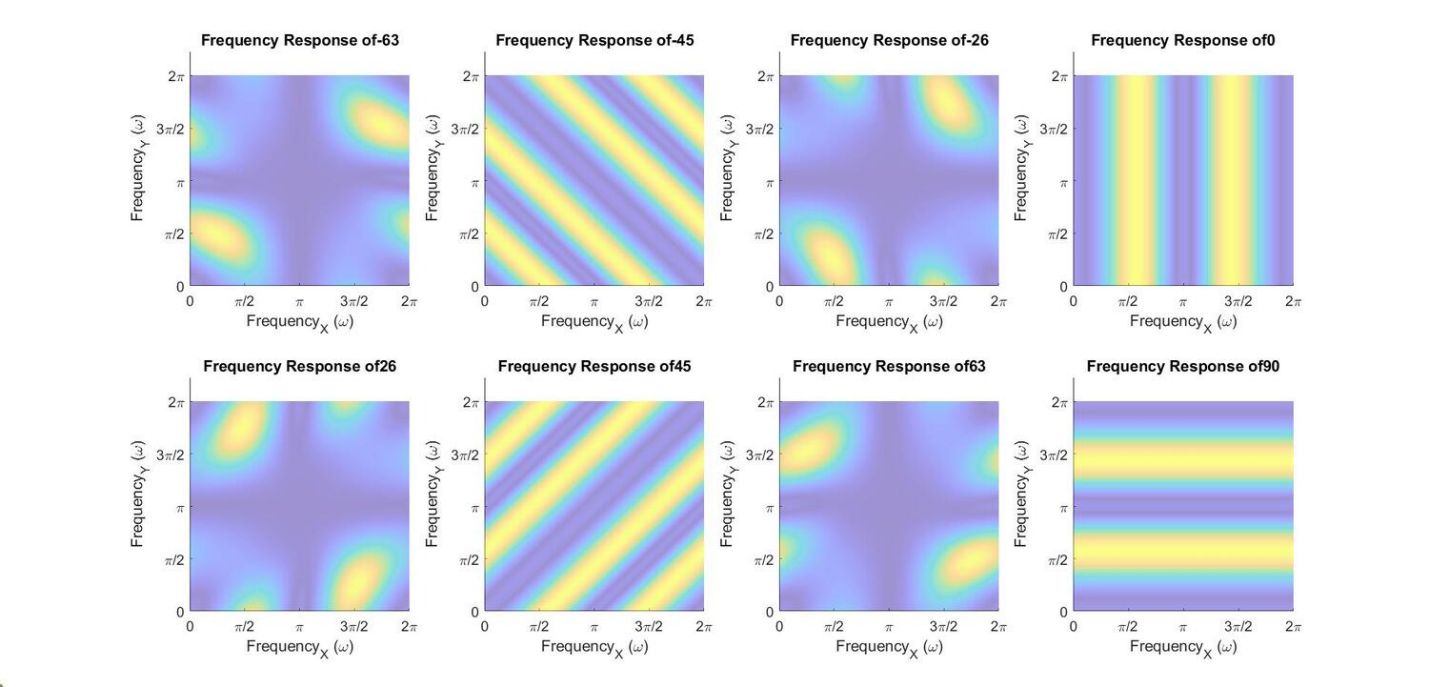


**S3 Fig.** **The proposed deep learning model without directional filters used for comparison.**


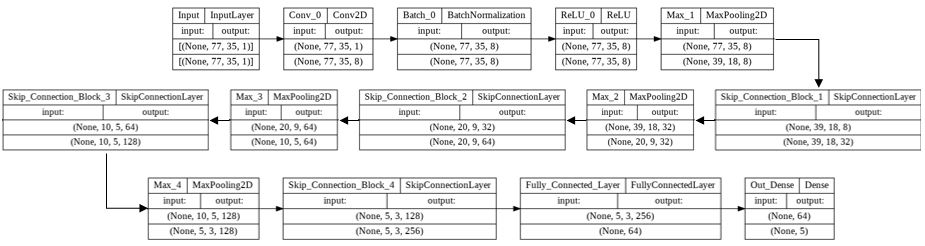

Supplement: S1 File — (DOCX) [file pone.0269198.s001.docx]
